# Microstructure-Property Regulation in a Large-Size Mg-9.4Gd-5.8Y-1Zn-0.5Zr Alloy by Differential Phase Electromagnetic Semi-Continuous Casting and Homogenization

**DOI:** 10.3390/ma19020282

**Published:** 2026-01-09

**Authors:** Yonghui Jia, Fangkun Ning, Yao Cheng, Yunchang Xin, Weitao Jia

**Affiliations:** 1 Key Laboratory of Light-Weight Materials, Nanjing Tech University, Nanjing 210009, China; 2 School of Mechanical Engineering, Taiyuan University of Science and Technology, Taiyuan 030024, China

**Keywords:** Mg-Gd-Y-Zn-Zr alloy, macroscopic segregation, differential phase electromagnetic semi-continuous casting, homogenization, solid solution strengthening, LPSO phase

## Abstract

Based on a novel semi-continuous casting mold with independent primary cooling regulation, a large-size Mg-9.4Gd-5.8Y-1Zn-0.5Zr alloy billet (Ø330 mm) was successfully fabricated via differential phase electromagnetic vibration casting. This process significantly improved microstructural homogeneity, with grain sizes ranging from 117 µm to 130 µm across the billet and elemental segregation of Gd and Y below 3%. Homogenization at 520 °C for 5 h effectively dissolved grain boundary eutectic phases; promoted diffusion of Gd, Y, and Zn into the α-Mg matrix; and stimulated the precipitation of fine LPSO lamellae. These microstructural improvements resulted in an excellent tensile strength of 208.4 MPa and elongation of 24.4%, demonstrating an optimal strength–ductility balance achieved through precise thermal processing.

## 1. Introduction

Magnesium (Mg) is the lightest constructional metal, making it an essential material for automotive, transportation, aviation, aerospace, etc. [1,2]. However, the poor mechanical properties (i.e., tensile and creep) of Mg alloys limit their applications [3,4]. Therefore, high-performance Mg alloys have gradually become essential materials for lightweight equipment in related fields, especially aviation and aerospace industries. It has been reported that the addition of rare-earth (RE) elements (i.e., Gd and Y) to Mg alloys can cause the precipitation strengthening effect and formation of a long-period stacking ordered (LPSO) phase to overcome the disadvantages [5,6,7,8], improving both room and elevated temperatures mechanical properties [9,10,11,12,13]. The addition of RE elements can enable magnesium alloys to meet higher mechanical properties while achieving weight reduction and expanding their applications. Therefore, the Mg-Gd-Y alloy system has become a promising alloy system [14], which usually has excellent mechanical properties at room temperature, even exceeding the commercial WE43 and QE22 alloys.

However, the high-performance Mg-Gd-Y alloy series often requires the addition of a higher content of Gd and Y. Srinivasan et al. [3] studied the hot-tearing characteristics of binary Mg-Gd (1–10, wt.%) alloys with an instrumented CRC mold, and it was found that the peak and least hot-tearing susceptibilities (HTS) of Mg–Gd binary alloys occur at 2 wt.% Gd and 10wt.% Gd, respectively. Han’s study [15] also found that the peak HTS of a Mg-Gd-(Y) alloy appears at 2 wt.%, and HTS of the alloy decreases with the increase in Gd content; in addition, he found that the alloy exhibits cold cracking tendency when Gd content exceeded 7 wt.%. Moreover, for the semi-continuous casting of large-size Mg-Gd-Y alloy ingot, due to the significant temperature difference along the radial direction of the ingot, the casting stress and macrosegregation of rare-earth elements are enormous. It leads to an evident tendency to crack, significantly limiting the preparation of large-sized casting billets. Casting cracks are severe problems in the semi-continuous casting process, especially when casting large-scale billets with high alloying-RE-element content.

Most research has mainly focused on the performance under laboratory conditions. Till now, little research has addressed the implementation of Mg-RE under industrial mass-production conditions, especially large-size high-RE Mg-alloy billets, which is also an important reason to limit their application. Hence, in the present investigation, the preparation and evaluation of a large-size Mg-Gd-Y-Zn-Zr alloy billet fabrication were carried out based on the differential phase electromagnetic semi-continuous casting technique.

## 2. Materials and Experiment Methods

### 2.1. Large-Size Ingot Preparation

**Figure 1:**
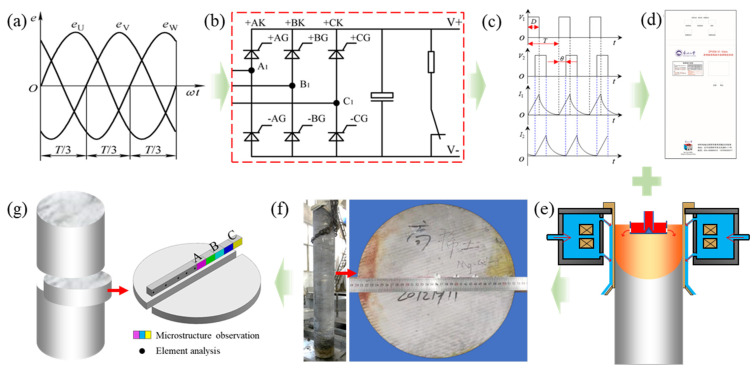
Schematic diagram of the preparation of the ingot with a diameter of Φ330 mm by differential-phase vibration electromagnetic field (DP-VMF) semi-continuous casting technique and samples for microstructure observation: (**a**) three-phase alternating current; (**b**) rectifier, filter, inverter, and control circuit; (**c**) differential phase vibration current; (**d**) DP-VMF generator system; (**e**) semi-continuous casting in the application of DP-VMF; (**f**) overall appearance and internal cross-section of the ingot with a diameter Φ330 mm; (**g**) A, B and C are sampling positions for microstructure observation and element analysis.

In the process of alloy melting, KJ-5 flux was added to the bottom of the crucible before alloy melting, and then the required amount of magnesium ingot, Mg_25_Gd, and Mg_30_Y intermediate alloy ingot was added. The raw ingots were heated to 730~740 °C to melt completely, and the melt was mechanically stirred for 3~5 min and then heated to 790 °C to add the required amount of Mg-30Zr intermediate alloy. When the temperature was raised to 805 °C, the mixture was mechanically stirred for 8 min, and argon-stirred for 7 min. At the same time, flux was added for refining. Finally, after standing at 805~810 °C for about one hour, the casting was started when the temperature was lowered to 760~770 °C. The casting process parameters are shown in Table 1. Figure 1 shows the schematic diagram of the preparation of the ingot with a diameter of Φ330 mm, by the differential phase vibration electromagnetic field (DP-VMF) semi-continuous casting technique, and samples for microstructure observation. It can be seen from Figure 1f that the surface quality of the billet was good, and there was no cold shut defect, subsurface crack extending inward at the edge of the ingot, nor defects, such as cracks and oxide inclusions, found in other areas of the cross-section of the ingot.

### 2.2. Homogenization Processing

In the as-cast Mg-Gd-based magnesium alloy system, factors such as dendritic segregation, non-equilibrium phases, and residual stress can reduce the alloy’s plasticity, which is detrimental to subsequent deformation processing. Therefore, a necessary homogenization treatment was required to improve the microstructure and mechanical properties of the magnesium alloy. To control experimental variables, wire cutting was used to sample the alloy at half the radius of the ingot (i.e., the 1/2R position). The set homogenization temperatures were 470 °C and 520 °C, with holding times of 5, 10, 15, and 20 h, respectively. After homogenization, the samples were immediately water-quenched to suppress the precipitation of supersaturated solid solutions.

### 2.3. Microstructural Observation and Mechanical Property Testing

All samples for microstructural observation were prepared using standard metallographic procedures. The etchant was composed of 70 mL alcohol, 4.2 g picric acid, 10 mL glacial acetic acid, and 10 mL distilled water. The polished surfaces were etched for 8~12 s. The etched samples were subsequently examined using a LECA-DMI5000M optical microscope (Leica Microsystems, Wetzlar, Germany) and a ZEISS Sigma 300 scanning electron microscope (SEM) (Carl Zeiss Microscopy, Oberkochen, Germany) equipped with an energy dispersive spectrometer (EDS) for microstructural observation and identification of secondary phases.

The grain size was measured by Image-Pro Plus v7 (IPP) software. The grain size and its distribution were evaluated by the frequency distribution method. It is shown that the measured grain size roughly follows the logarithmic normal distribution function, which is expressed as follows:

(1) $$P=\frac{1}{Sd\sqrt{2\pi }}\exp\left[-\frac{\ln^{2}(d/d_{m})}{2S^{2}}\right]$$

The composition of the ingot was determined by an inductively coupled plasma analyzer (ICP, Perkin Elmer, Plasma-400, Waltham, MA, USA). The evaluation of the segregation degree was calculated as follows:

(2) $$\eta_{i}=\frac{x_{i}-\sum x_{i}/n}{\sum x_{i}/n}\times 100\%$$

$\eta_{i}$ is the segregation rate, $x_{i}$ the element content of the analysis point, and i the number of analysis points.

Room-temperature tensile tests were performed on rectangular specimens whose longitudinal axis was aligned parallel to the casting direction. Each specimen had an initial gauge length of 20 mm, a width of 10 mm, and a thickness of 2 mm. Testing was conducted on a SHIMADZU AGS-X universal electromechanical testing machine (Shimadzu Corporation, Kyoto, Japan) at a constant crosshead speed of 1 mm·min^−1^. To ensure data accuracy and reproducibility, at least three specimens were tested for each condition.

## 3. Results and Discussion

### 3.1. Grain Size Evaluation

**Figure 2:**
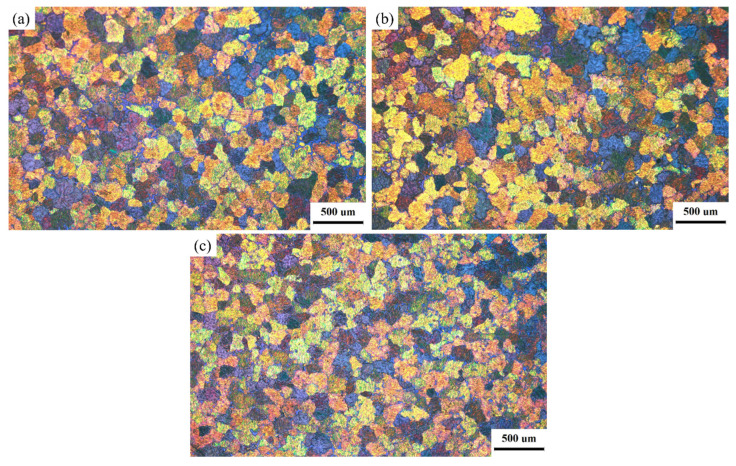
Macrostructures of the ingot fabricated by DP-VMF semi-continuous casting at the center (**a**), R/2 (**b**), and edge (**c**) of the billet.

**Figure 3:**
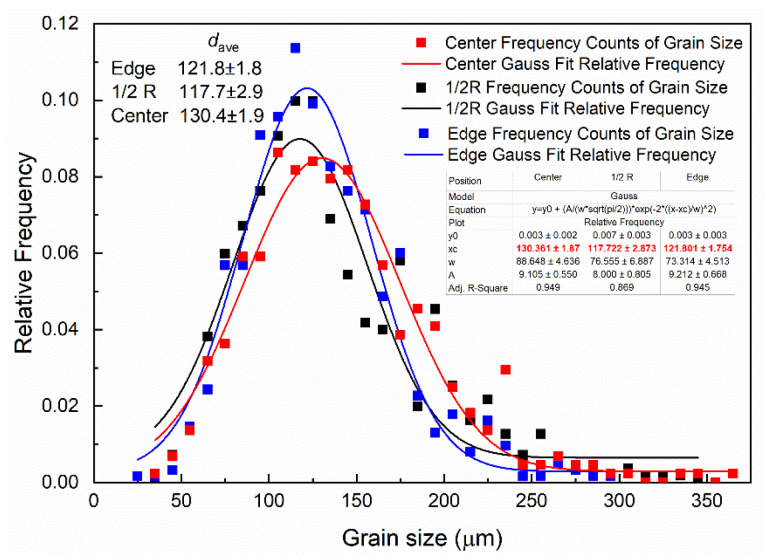
Statistics of the grain size of the ingot.

Figure 2 shows the macroscopic structure of the ingot. From the center of the casting billet to the edge, it can be seen that there is a fine and uniform equiaxial crystal structure, which is significantly more refined than that of the AZ31B alloy [2]. The reason for this is that, in addition to the structure refinement effect of the electromagnetic field (EMF) itself, Y and Zr elements in the alloy also play a role in refining the structure during solidification [12]. The statistical results of grain size shown in Figure 3 demonstrate that the grain size distribution inside the ingot exhibits good uniformity. The grain sizes from the center to the edge of the ingot are about 121 µm, 117 µm, and 130 µm, with a range of 13 µm, and the maximum deviation of the grain size is only 6.5% ((130 − 121)/130 × 100% ≈ 6.5%).

Table 2 shows the Mg-Gd-Y alloy ingot with high rare-earth content prepared by a semi-continuous casting process in this study, alongside alloys reported in the literature. It can be seen that with the increase in ingot diameter, the solidification structure of the billet is significantly coarsened, and the uniformity worsens. At the same time, applying an EMF can dramatically refine the microstructure and improve the uniformity of grain size distribution. For the Φ190 mm billet cast by hot-top mold, the surface of the ingot formed a stripe with a depth of about 3 mm due to the friction between the insulation material and the billet, resulting in poor surface quality [16]. However, the grain size uniformity of the Φ315 mm ingot prepared by the conventional semi-continuous casting process was poor, and there were macro casting defects in the edge area of the ingot [17], which would significantly increase the turning amount of the billet surface. However, the grain size of the ingot with a diameter of 330 mm prepared by the new mold and DP-VMF was significantly reduced, and the grain size distribution uniformity and internal defect control were improved considerably.

### 3.2. Macrosegregation

**Figure 4:**
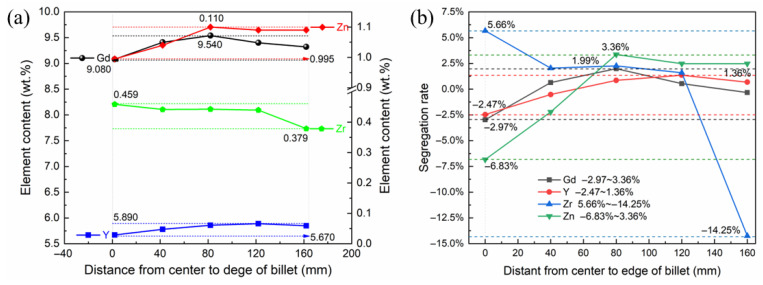
Element content and macro-segregation of billets. (**a**) Element content; (**b**) Segregation rate.

ICP analysis was performed on five samples with equal spacing from the outside to the inside along the radius of the ingot, and the results are shown in Table 3. In accordance with the results in Table 3, the content and segregation rate of the main elements along the radial direction of the ingot were analyzed, as shown in Figure 4. The results showed that the segregation rates of Gg, Y, Zr, and Zn were −2.89~2.03%, −2.41~1.38%, −12.39~6.10%, and −6.40~3.48%, respectively. The segregation rates of Gd and Y were controlled within 3%, and the distribution uniformity of rare earth elements in the cross-section of the ingot was good. In addition, it can be seen that the content of the Zr element gradually increased from the edge to the center. Since the 1/2 radius was close to the liquid outlet of the shunt and belonged to the continuous high-temperature region, the content was slightly higher than the 3/4 radius from the edge.

### 3.3. Effect of Homogenization on Microstructure

**Figure 5:**
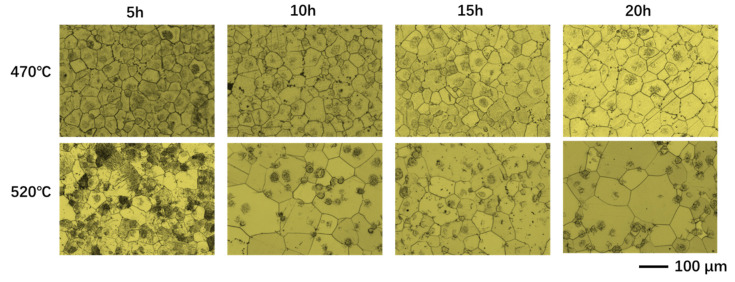
Micrographs of billets at solution treatment temperatures of 470 °C and 520 °C with different holding times.

**Figure 6:**
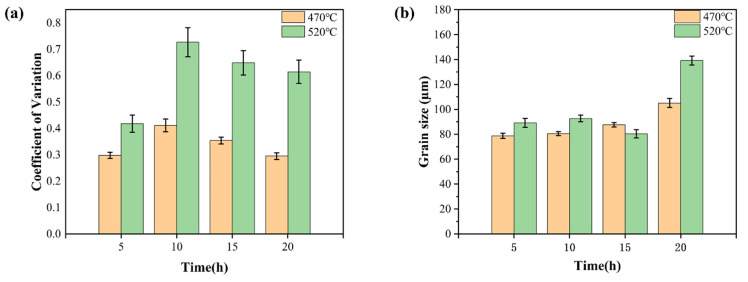
Coefficient of variation (**a**) and average grain size (**b**) of billets at solution treatment temperatures of 470 °C and 520 °C with different holding times.

As shown in Figure 5, at the solution treatment temperature of 470 °C, only minor recrystallization occurred after 5 h of holding, with pronounced recrystallization characteristics appearing only after 10 h. In contrast, at 520 °C, significant recrystallization was observable after just 5 h of holding, and the number of secondary recrystallizations increased markedly during the 10 to 15 h period. Compared to the 470 °C condition, grain growth and recrystallization nucleation proceeded more rapidly at 520 °C, enabling significant microstructural changes within a shorter timeframe. This indicates that billets exhibited greater sensitivity to the solution treatment regimen at 520 °C than at 470 °C. As shown in Figure 6, under the solution treatment conditions of 470 °C-5 h and 520 °C-5 h, both the average grain size and dispersion index reached their optimal values at each respective temperature. Specifically, the average grain size at 520 °C-5 h was 89.1 µm, representing a 13.1% increase compared to the 78.7 µm observed at 470 °C-5 h. Considering both the sensitivity to homogenization conditions and grain size characteristics, it can be concluded that the 520 °C-5 h condition yielded superior grain size, size dispersion, and responsiveness to homogenization conditions.

**Figure 7:**
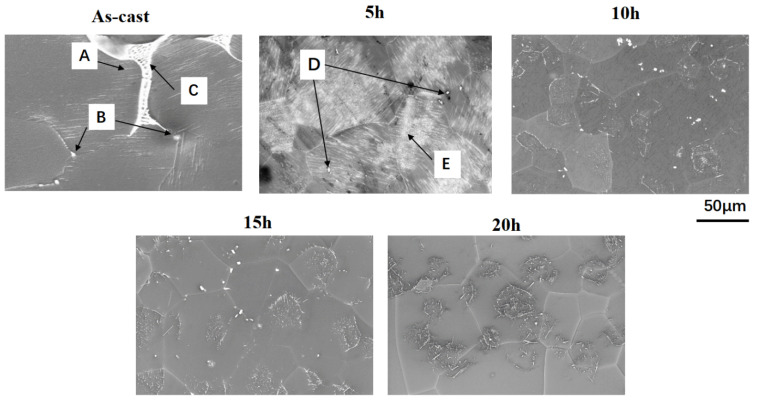
BSE images of the ingot in the as-cast state and after different holding times at 520 °C. A—lamellar LPSO phase; B—blocky Mg-RE phase; C—bright eutectic network (Mg_24_(Gd, Y, Zn)_5_); D—dissolved and transformed blocky phase after homogenization; E—fine lamellar LPSO phase precipitated within grains.

**Figure 8:**
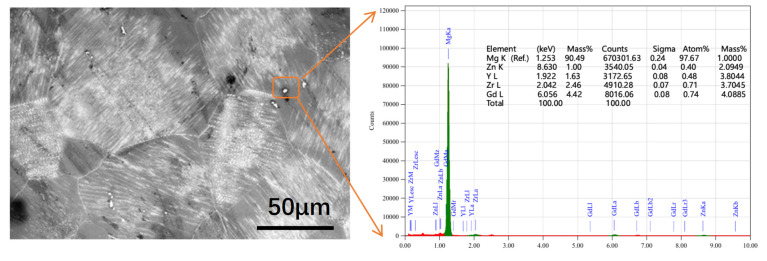
BSE morphology and EDX analysis of Mg-9.4Gd-5.8Y-1Zn-0.5Zr alloy after homogenization at 520 °C for 5 h under solution treatment conditions.

As shown in Figure 7, the as-cast alloy is mainly composed of a Mg matrix, with bright white intergranular network phases, and gray blocky phases at the grain boundaries. In accordance with previous studies, in the as-cast Mg-Gd-Y-Zn-Zr alloy, the lamellar phase adjacent to the eutectic network is likely the LPSO phase (Figure 7A); the punctate or blocky phases along the grain boundaries are presumably Mg–Re phases formed by the segregation of Gd, Y, Zn, and Zr (Figure 7B); and the bright white reticular phase is inferred to correspond to Mg_24_(GdYZn)_5_ (Figure 7C) [20]. Under the solution treatment conditions of 520 °C-5 h, the continuously distributed network eutectic gradually dissolves, with a significant reduction in the amount of coarse second phase at grain boundaries, and partial transformation into a uniformly distributed blocky phase (Figure 7D) [21]. As more rare-earth elements solid-solve into the matrix, fine lamellar LPSO phases (Figure 7E) precipitate within grains. Their orientation is consistent within the same grain but differs between distinct grains [22]. As shown in Figure 8, the dispersed block-like phase at grain boundaries is formed by the segregation of elements, such as Gd, Y, Zn, and Zr. Studies have shown that this indicates its primary composition is Mg_12_Zn(Gd, Y) [20]. Concurrently, the fine, densely packed lamellar phase has been confirmed to exhibit an LPSO structure. For Mg alloys, the primary plastic deformation mechanisms at room temperature are {0001} basal slip and tensile twinning; however, due to the grain structure being saturated with the LPSO phase, the twinning process in this slow-cooling alloy is significantly restricted [23]. The LPSO phase possesses a higher elastic modulus and hardness than the *α*-Mg matrix, and {0001} basal slip dominates its plastic deformation [24]. Consequently, the LPSO phase acts as a fiber reinforcement, effectively strengthening the alloy [25]. At 520 °C-5 h, the grain interior filled with LPSO phase was the key factor in the alloy’s 49.6% increase in elongation. Previous studies have demonstrated that coarse reticular eutectic structures at grain boundaries induce brittle fracture, reducing alloy strength and ductility [26]. Following homogenization, approximately 5.5% of the coarse reticular eutectic in the as-cast state transformed into an about 0.3% massive phase, while substantial LPSO phase precipitated within grains and rare-earth elements further dissolved into the *α*-Mg matrix [27]. Furthermore, as reported by Wei et al., dislocations can be trapped by solute atoms to form a Cottrell atmosphere, requiring additional stress to drive dislocation motion and thereby enhancing yield strength [28]. Consequently, the increased rare-earth element solid solution in the α-Mg matrix strengthens the solid solution strengthening effect, leading to improved yield strength and tensile strength of the billet. As shown in Figure 7, the proportion of blocky phase increases with extended holding time, rising from 0.3% at 5 h to 0.8%, 0.8%, and 0.6% at 10, 15, and 20 h, respectively. The blocky phase precipitates near grain boundaries during solid solution treatment [29]. Excessively long holding times increase the proportion of this precipitate phase. Concurrently, Figure 7 and Figure 8 indicate that prolonged holding times cause grain growth. The combination of increased precipitate phase and grain coarsening due to excessive homogenization time constitutes the primary cause of reduced alloy strength. In summary, the solid solution condition of 520 °C-5 h achieved complete dissolution of coarse eutectic phases and enhanced the solid-solution degree. Concurrently, strengthening phases (LPSO) precipitated within grains. The combined effects of solid-solution strengthening and LPSO strengthening optimized the billet’s strength and plasticity.

**Figure 9:**
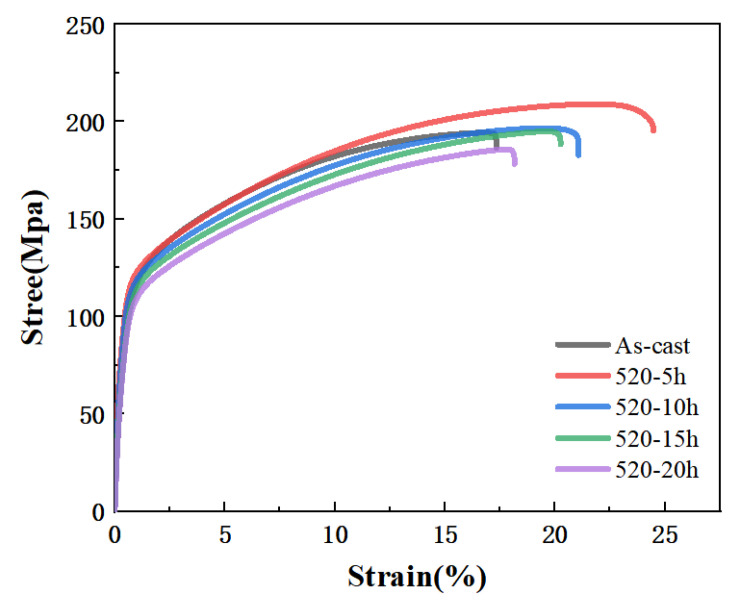
Tensile properties of the alloy in the as-cast condition and after solution treatment at 520 °C with different holding times.

As the holding time increases, the solution treatment process exhibits distinct stages. In the initial stage (e.g., within 5 h at 520 °C), the solubility of rare earth elements increases significantly, as evidenced by the dissolution of the non-equilibrium eutectic phase and the precipitation of fine lamellar LPSO phases within grains. However, when the soaking time exceeds 5 h (e.g., 10, 15, or 20 h), the solubility approaches saturation, and the microstructure begins to deteriorate. On one hand, the grains grow notably, as shown in Figure 6b, the average grain size increases from 89.1 µm at 5 h to approximately 110 µm at 10 h at 520 °C, reducing the grain boundary strengthening effect. On the other hand, the supersaturated solid-solution precipitates coarsen blocky phases (mainly Mg_12_Zn(Gd, Y)) near grain boundaries, with their area fraction rising from 0.3% at 5 h to 0.8% at 10 h (Figure 7). These coarse precipitates exhibit weaker interfacial bonding and may act as crack initiation sites. Additionally, prolonged holding time leads to partial coarsening or morphological changes in the LPSO phase, weakening its fiber-reinforcement effect. Therefore, while initial soaking enhances solid-solution and fine-phase precipitation, excessive time induces grain growth and precipitate coarsening, both contributing to the decline in alloy strength (as reflected in the reduced tensile strength for treatments beyond 10 h in Figure 9). This elucidates why 520 °C-5 h represents the optimum condition: it maximizes solid-solution and LPSO strengthening while avoiding microstructural degradation.

Figure 9 and Table 4 indicate that the tensile test results demonstrate optimal mechanical properties were achieved for the billets under the 520 °C-5 h condition. Compared to the as-cast state, tensile strength increased from 194 MPa to 208.4 MPa, representing a 7.4% increase; yield strength rose from 105.8 MPa to 109.6 MPa, a 3.6% increase; and elongation significantly improved from 17.3% to 24.4%, a substantial 41% increase. The strength improvement primarily resulted from the solid solution strengthening effect of rare-earth elements and the fiber reinforcement effect of fine LPSO phases. The significant increase in elongation was closely related to the dissolution of the reticular eutectic phase and the reduction in grain boundary brittleness. In summary, 520 °C-5 h can be considered the optimal homogenization treatment for balancing strength and ductility.

## 4. Conclusions

A high-strength and tough magnesium alloy ingot with the composition Mg-9Gd-5.6Y-1Zn-0.5Zr (by weight percentage), measuring Φ330 mm in diameter, was successfully produced using a novel electromagnetic semi-continuous casting mold. The mold incorporated an adjustable primary cooling system and a differential vibrating electromagnetic field. The ingot exhibited no cracks on its surface or interior. It also showed uniform grain size and rare-earth element distribution. Grain sizes from the center to edge were about 121 µm, 117 µm, and 130 µm, with a maximum deviation of only 6.5%. Segregation rates of gadolinium (Gd) and yttrium (Y) were controlled within ±3%.

In Mg-Gd-Y-Zn-Zr alloys, the homogenization regimen at a solution treatment temperature of 520 °C is more sensitive than that at 470 °C. The optimal homogenization treatment for the alloy is a 5 h soak at 520 °C. This homogenization condition effectively dissolves the network eutectic in the as-cast Mg-Gd-Y-Zn-Zr alloy, dissolves rare-earth elements into the *α*-Mg matrix, reduces segregation, and enhances solution strengthening. The formation of fine, dense LPSO phases within grains acts as fiber reinforcement, synergistically enhancing both strength and ductility. Under the dual strengthening effects of solution treatment and the LPSO precipitation phase, yield strength, tensile strength, and elongation increased by 7.4%, 3.6%, and 41%, respectively, compared to the as-cast state.

## Figures and Tables

**Table 1:** Semi-continuous casting parameters for the ingot preparation.

Casting Temperature	Casting Speed	Current	Frequency	Duty Cycle
760~770 °C	31~35 mm·min^−1^	90~120 A	20 Hz	20%

**Table 2:** The preparation of Mg-Gd-Y high-RE alloy billets in this work and reported in the literature.

Alloy Composition (wt.%)	Billet Size (mm)		Technique	Grain Size	Ref.
	Diameter	Length		µm	
Mg-9.3Gd-3Y-1.5Zn-0.6Zr	190	1000	^1^ Semi-continuous + EMF	28~35	[16]
Mg-8Gd-3Y-0.5Zr	110	800	Semi-continuous	~33	[18]
Mg-8.2Gd-3.8Y-1.0Zn-0.4Zr	280	2940	Semi-continuous	78~126	[19]
Mg-8Gd-4Y-1Zn-Mn	315	2410	Semi-continuous	102~306	[17]
Mg-9.4Gd-5.8Y-1Zn-0.5Zr	330	2100	^2^ Semi-continuous + DP-VMF	117~130	This paper

^1^ Semi-continuous + EMF—semi-continuous casting + conventional EMF; ^2^ Semi-continuous + DP-VMF—this work applies semi-continuous casting + novel DP-VMF.

**Table 3:** Element contents at different positions in the cross-section of billets (mass fraction, %).

Analysis Positions	Gd	Y	Zr	Zn	Mg
1# (center)	9.080	5.670	0.459	0.995	Bal.
2# (3/4 radius)	9.410	5.780	0.442	1.040	Bal.
3# (1/2 radius)	9.540	5.860	0.443	1.100	Bal.
4# (1/4 radius)	9.400	5.890	0.440	1.090	Bal.
5# (edge)	9.320	5.850	0.379	1.090	Bal.

**Table 4:** Tensile properties of the alloy in Figure 9.

Condition	YS (MPa)	UTS (MPa)	El (%)
As-cast	105.8	194.0	17.3
520 °C-5 h	109.6	208.4	24.4
520 °C-10 h	~108.0	~200.0	~22.0
520 °C-15 h	~105.0	~195.0	~20.0
520 °C-20 h	~103.0	~190.0	~18.0

## Data Availability

The original contributions presented in this study are included in the article. Further inquiries can be directed to the corresponding authors.
